# Supplementary material for: The Conversion of Superoxide to Hydroperoxide on Cobalt(III) Depends on the Structural and Electronic Properties of Azole-Based Chelating Ligands
Source: Molecules. 2022 Sep 28;27(19):6416. doi: 10.3390/molecules27196416 (PMC9571172; doi:10.3390/molecules27196416)
Supplement: Supplementary file 1 [file molecules-27-06416-s001.zip › molecules-1915800-supplementary.pdf]

Supplementary Materials *for:*

The Conversion of Superoxide to Hydroperoxide on Cobalt(III) Depends on the Structural and Electronic Properties of Azole-Based Chelating Ligands

Toshiki Nishiura [1], Takehiro Ohta [2,†], Takashi Ogura [2,‡], Jun Nakazawa [1], Masaya Okamura [1] and Shiro Hikichi [1,*]

[1] Department of Material and Life Chemistry, Faculty of Engineering, Kanagawa University, 3-27-1 Rokkakubashi, Kanagawa-ku, Yokohama 221-8686, Japan
[2] Department of Life Science, University of Hyogo, Ako-gun, Hyogo 678-1297, Japan
* Correspondence: hikichi@kanagawa-u.ac.jp; Tel.: +81-45-481-5661
† Current address: Department of Applied Chemistry, Sanyo-Onoda City University, Yamaguchi 756-0884, Japan.
‡ Passed away on 23 July 2017.

List of contents

Figure S1: Simulated absorption spectra
Figure S2: Mulliken spin density and spin density distribution of the superoxido complex.
Figure S3: Charge distribution of the superoxido complex and the hydroperoxido complex obtained by the natural population analysis.
Figure S4: Selected molecular orbitals of the superoxido complex
Figure S5: Selected molecular orbitals of the hydroperoxido complex.

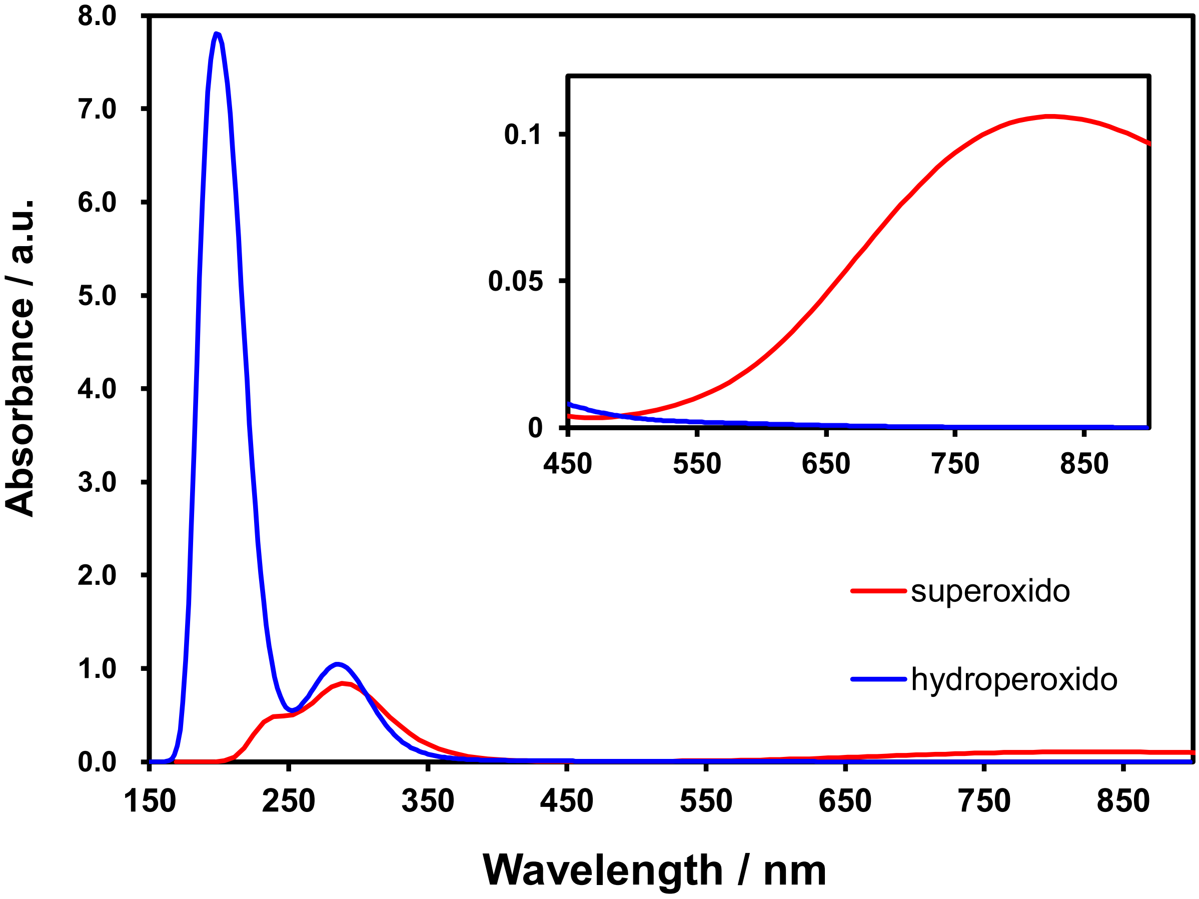

Figure S1. Simulated absorption spectra.

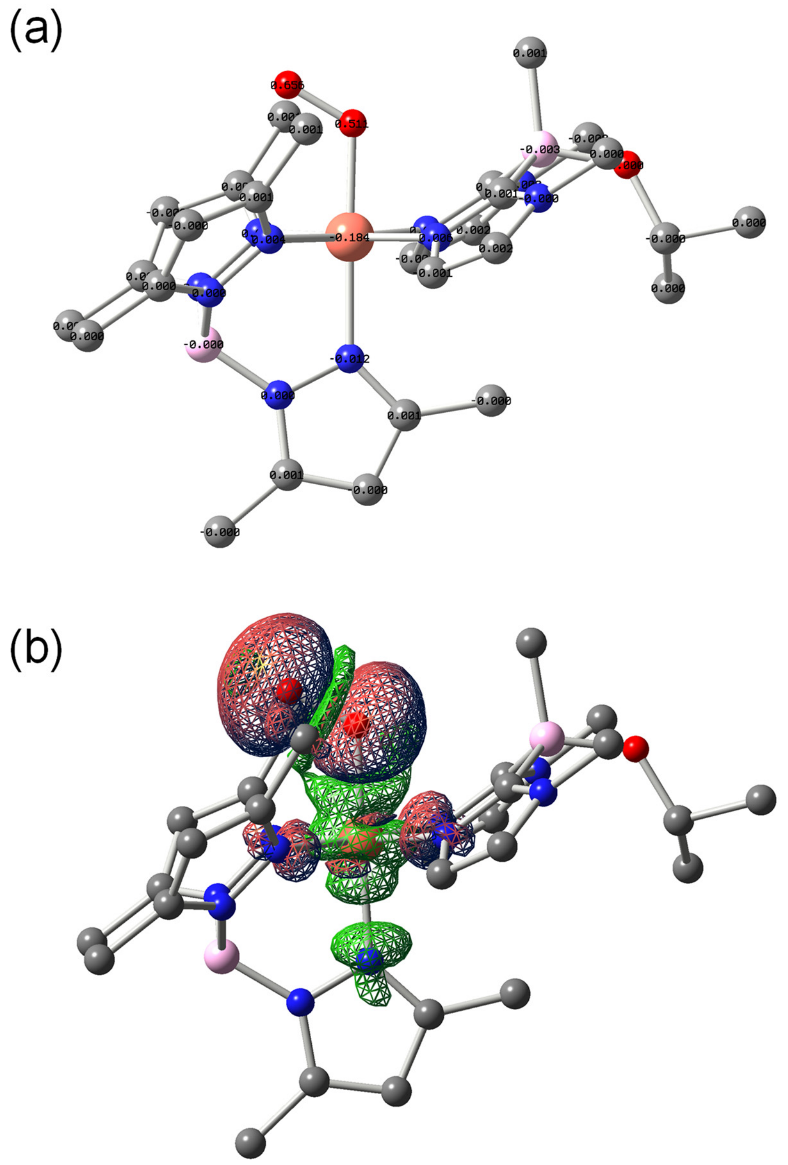

Figure S2. Mulliken spin density (a) and spin density distribution (b) of the superoxido complex.

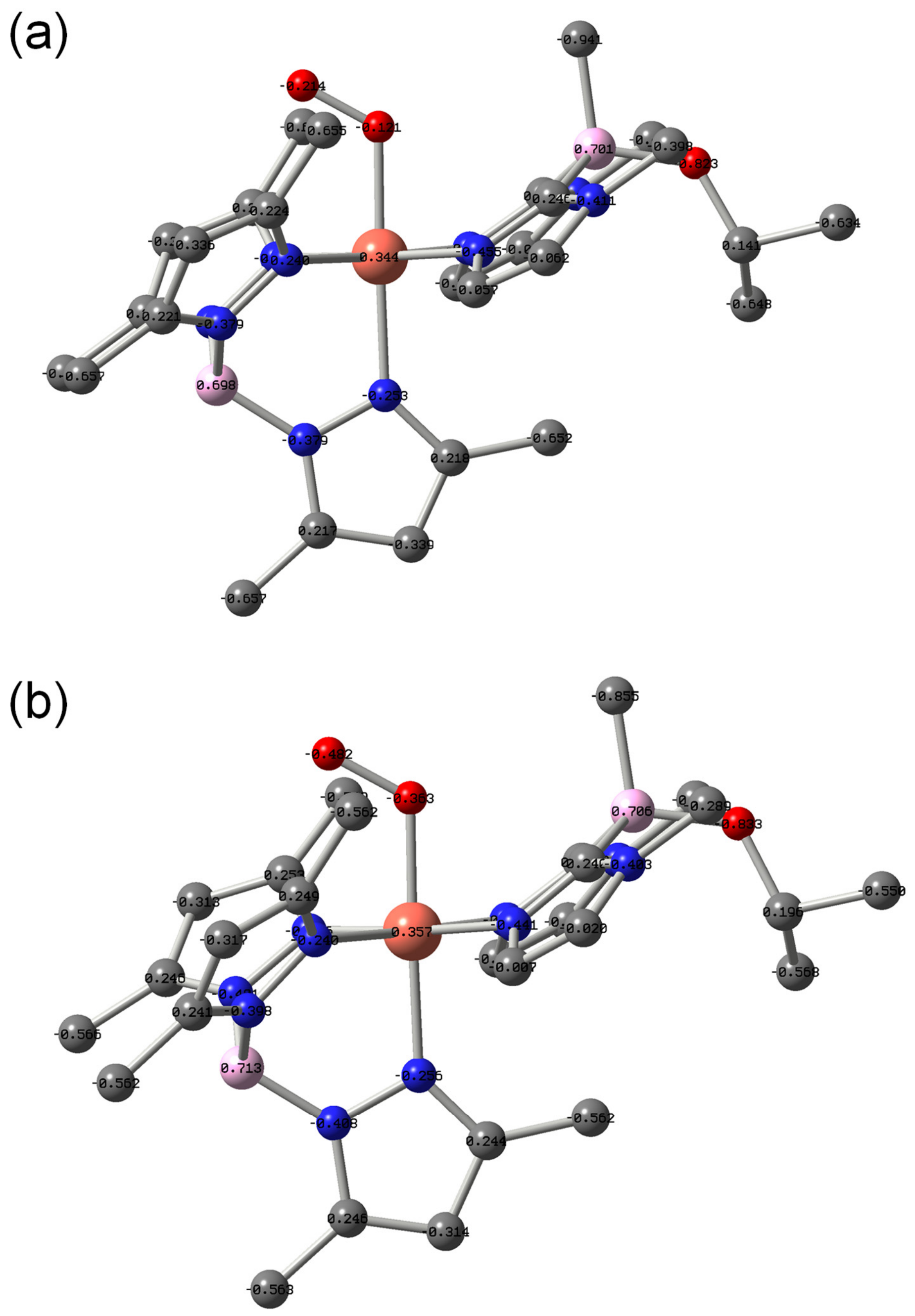

Figure S3. Charge distribution of the superoxido complex (a) and the hydroperoxido complex (b) obtained by the natural population analysis.

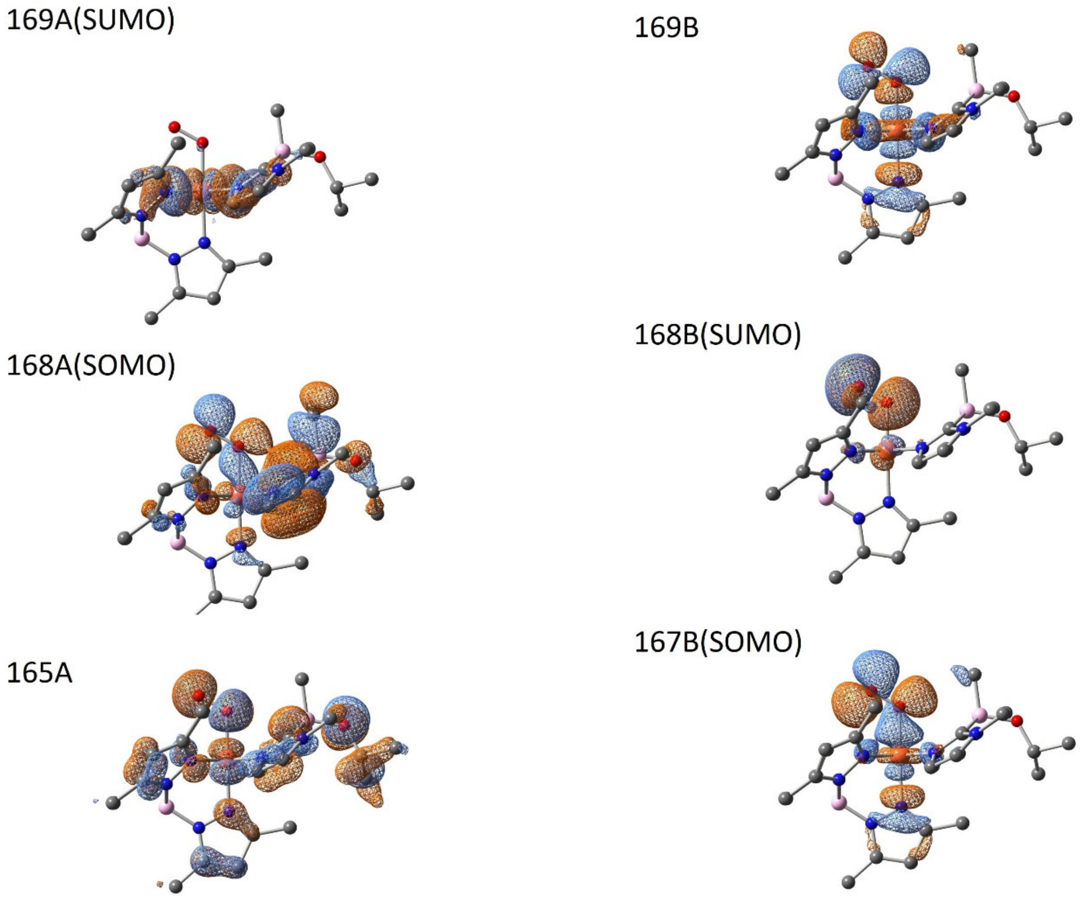

Figure S4. Selected molecular orbitals of the superoxido complex

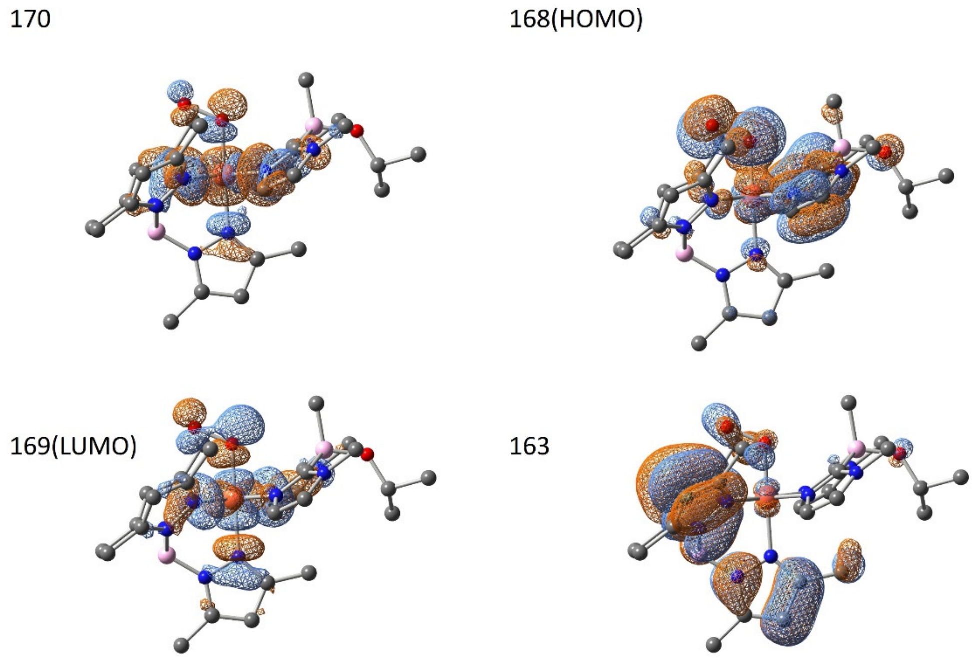

Figure S5. Selected molecular orbitals of the hydroperoxido complex.

List of the references for the DFT calculation [23–29].
